# Comprehensive comparison of neonate and adult human platelet transcriptomes

**DOI:** 10.1371/journal.pone.0183042

**Published:** 2017-08-16

**Authors:** Eva Caparrós-Pérez, Raúl Teruel-Montoya, Mª José López-Andreo, Mª Carmen Llanos, José Rivera, Verónica Palma-Barqueros, Jose E. Blanco, Vicente Vicente, Constantino Martínez, Francisca Ferrer-Marín

**Affiliations:** 1 Hematology and Medical Oncology Department. Centro Regional de Hemodonación, Morales Meseguer University Hospital, IMIB-Arrixaca, Murcia, Spain; 2 Red CIBERER (CB15/00055), Instituto Carlos III, Madrid, Spain; 3 University of Murcia, Murcia, Spain; 4 Obstetrics and Gynecology Service, Virgen de la Arrixaca Clinical Hospital, Murcia, Spain; 5 Grado de Medicina, Universidad Católica de Murcia (UCAM), Murcia, Spain; Huazhong University of Science and Technology, CHINA

## Abstract

Understanding the underlying mechanisms of the well-substantiated platelet hyporeactivity in neonates is of interest given their implications for the clinical management of newborns, a population at higher bleeding risk than adults (especially sick and preterm infants), as well as for gaining insight into the regulatory mechanisms of platelet biology. Transcriptome analysis is useful in identifying mRNA signatures affecting platelet function. However, human fetal/neonatal platelet transcriptome analysis has never before been reported. We have used mRNA expression array for the first time to compare platelet transcriptome changes during development. Microarray analysis was performed in pure platelet RNA obtained from adult and cord blood, using the same platform in two independent laboratories. A high correlation was obtained between array results for both adult and neonate platelet samples. There was also good agreement between results in our adult samples and outcomes previously reported in three different studies. Gene enrichment analysis showed that immunity- and platelet function-related genes are highly expressed at both developmental stages. Remarkably, 201 genes were found to be differentially expressed throughout development. In particular, neonatal platelets contain higher levels of mRNA that are associated with protein synthesis and processing, while carrying significantly lower levels of genes involved in calcium transport/metabolism and cell signaling (including *GNAZ*). Overall, our results point to variations in platelet transcriptome as possibly underlining the hypo-functional phenotype of neonatal platelets and provide further support for the role of platelets in cellular immune response. Better characterization of the platelet transcriptome throughout development can contribute to elucidate how transcriptome changes impact different pathological conditions.

## Introduction

Platelets play critical roles in normal hemostatic processes, but also as regulators or mediators of angiogenesis, defense against microorganisms, or inflammation in pathologic conditions [1]. Generated from fragmentation of megakaryocyte (MK) cytoplasm, platelets are anucleate blood cells that retain thousands of MK-derived transcripts and contain a rough endoplasmic reticulum, polyribosomes, translation initiation and termination factors, and regulators, such as microRNAs, all of which are prerequisites to maintaining functionally intact protein translational capabilities [2]. Transcriptome analysis is useful to identify functional changes in platelet biology. Indeed, thanks to these genomic techniques, mRNA expression profiling has been associated with cardiovascular disease [3], clonal *vs*. reactive thrombocytosis [4], and with platelet reactivity [5], among others. In fact, mRNA expression profiling has recently been used to identify the molecular basis for racial differences in platelet reactivity [6].

Ontogenic changes are well known in erythropoietic cells or macrophages [7], but little understood in the MK-platelet lineage. Over the last decade, it has become clear that fetal/neonatal and adult megakaryocytes have substantial biological differences [8, 9] and produce platelets with different functionality [10].

Full-term neonates exhibit platelet hyporeactivity in response to physiological agonists, which is more noticeable at lower gestational ages [11–15]. This neonatal platelet hyporeactivity is currently viewed as part of a delicate balance in the neonatal hemostatic system, somewhat more robust than adults, rather than as a developmental deficiency. Changes during development affect platelet reactivity, as well as many other aspects of platelet biology. Thus, a recent study has demonstrated that neonatal murine platelets have a longer lifespan than adult platelets in circulation and this is how neonates, whose hemostatic system volume is constantly growing, can maintain platelet counts without increasing platelet production [16].

The analysis of fetal/neonatal platelet transcripts may be clinically relevant not only for neonates. Therefore, interindividual variations in platelet reactivity in the normal population exist and might play an important role in pathological situations, such as stroke or hemorrhage. Given that neonates display extreme variance in platelet reactivity compared to adults, gene expression profiling of neonatal *vs*. adult platelets represents an ideal model to investigate the regulatory mechanisms of platelet function and to identify new therapeutic targets.

In the present study, we provide the first comprehensive comparison of neonate and adult human platelet transcriptomes. We performed mRNA microarray techniques in two independent, pure platelet, RNA pools from umbilical cord blood and adult peripheral blood using platforms from two independent laboratories to conduct this initial platelet transcriptome analysis throughout human development.

## Materials and methods

### Blood samples

The study was approved by the institutional review board at Virgen de la Arrixaca Clinical Hospital and written informed consent was obtained from all subjects in accordance with the Declaration of Helsinki. Blood samples from the umbilical cords of healthy full-term neonates and healthy adult peripheral blood were drawn with a 21-gauge needle and collected in 3.2% sodium citrate tubes (0.105 M buffered Citrate, BD, Plymouth, UK). The samples for both array and validation cohort were collected between December 2011 and February 2013.

### Platelet preparation

Blood samples were centrifuged (140 x *g*, 15 min at 22°C) to obtain platelet-rich plasma (PRP). Leukodepleted platelets from PRP were purified by filtration (PL1BE, Haemonetics, Braintree, MA) [17], followed by magnetic cell separation using Dynabeads CD45-coated beads (Miltenyi Biotec, Madrid, Spain) [18]. Erythrocytes were lysed by 1-min hypotonic shock with 0.2% saline. The CD45 immunodepleted platelet suspensions were separated into two aliquots, centrifuged at 6,000 x g and platelet pellets stored at -80°C until RNA and protein isolation.

### Western blot

Extracted platelet proteins were separated on SDS-PAGE gel under denaturing conditions. Blots were incubated with anti-GNAZ (sc-388, Santa Cruz Biotechnology, California, USA) primary antibody. Immunoreactivity was revealed by incubating with HRP conjugated anti-rabbit secondary antibody (NA9340, GE Healthcare Life Sciences, UK) and was detected by chemilumiscence.

### RNA isolation and leukocyte contamination analysis

Total RNA was isolated from ultrapure platelets with TRIzol reagent (Thermo Fisher Scientific) according to the manufacturer’s instructions. RNA concentration was measured using the NanoDrop 2000 spectrophotometer (Thermo Fisher Scientific) and RNA integrity was evaluated with the Agilent 2100 bioanalyzer (Agilent Technologies, Santa Clara, CA).

Residual leukocyte contamination in platelet preparations was quantified by qRT-PCR with *PTPRC-* (Protein Tyrosine Phosphatase Receptor Type C or *CD45*) specific primers [19], while platelet presence was confirmed using specific *ITGA2B* primers (ITGA2B-F: GAATGGCCCCTGCTGTCGTG; ITGA2B-R: ACGTCATCTTCCCCACAGTC).

### Microarray processing

Two pools (platelet RNA from 4 newborns or 4 adults) were sent to LC Sciences laboratories (Houston, TX) (referred to as array A). Another pair of pooled platelet RNA, from 2 newborns or 2 adults was sent to the central Molecular Biology Service of the University of Murcia (Murcia, Spain) (referred to as array B). Both arrays were performed with GeneChip Human Genome U133 Plus 2.0 Array (Affymetrix, Santa Clara, CA) containing 54,675 probes covering 23,800 genes. For each microarray, 350 ng of total RNA were processed according manufacturer´s indications (PN 703210, Rev1, Affymetrix, Santa Clara, CA).

### Microarray data, statistical, and pathway analysis

Raw data has been deposited at Gene Expression Omnibus (GEO) under accession number GSE94292. Quality controls of microarray data from arrays A and B were tested and raw data gene expression (.cel files) were normalized and summarized using the RMA function [20] of the ACCC Software [Affymetrix Gene Expression Console (Affymetrix, Santa Clara, CA)]. Statistical and pathway analysis of data were conducted using Partek Genomics Suite 6.6 and Partek Pathway (Partek Incorporated, St Louis, MO) and TAC Software (Affymetrix Transcriptome Analysis Console 3.0, Affymetrix, Inc). An ANOVA was executed to generate a list of genes displaying a differential expression pattern between adult and neonatal platelet pools. The cutoff applied to the ANOVA results was >2-fold change in gene expression at a significance level of p<0.05. Statistical and pathway data analysis were carried out with Partek Genomics Suite 6.6, operating in R-programming language.

Hierarchical clustering of differentially expressed genes was completed considering the expression of each gene standardized to mean 0 and standard deviation of 1 [21]. Gene enrichment analyses were performed using the PANTHER Overrepresentation Test (release 20160715) [22] and the GO biological process completed the analyses as the annotation data set (http://geneontology.org). Bonferroni test was used for multiples testing. *P*-values of <0.05 or <0.01 established significance for high and differential platelet gene expression analysis, respectively.

Data robustness was assessed by examining concordance between array A and array B density plots, based on the signal intensity for each probe and Venn diagrams representing the top 1,000 expressed genes created by GraphPad (GraphPad Software, Inc. La Jolla, CA) and SUMO (OncoExpress Software, Heidelberg University, Heidelberg, Germany)], respectively.

### Validation of microarray data by qRT-PCR

The validation cohort included 16 neonates and 16 adults. Selected genes were used to validate microarray results by qRT-PCR. Retro-transcription reaction was performed using 150 ng of total RNA for each sample, according to the manufacturer’s instructions (SuperScript III First Strand, Thermo Fisher Scientific). Taqman Premix Ex Taq (Takara Bio Inc.) and a commercial probe for *GNAZ* (Hs00157731_m1), *GNB5* (Hs00272529_m1), *RANBP10* (Hs00398714_m1), *PIK3CG* (Hs00932390_m1), *RPL32* (Hs00851655_g1), *PRDX2* (Hs00853603_s1), and *ACTB* (Hs99999903_m1) Taqman Gene Expression Assay (Thermo Fisher Scientific) and SYBR *Premix Ex Taq* (Takara Bio Inc.) with *ADRA2A-* and *ACTB-*specific primers (ADRA2A-F: CGACCAGAAGTGGTACGTCA; ADRA2A-R: TAGATGCGCACGTAGACCAG; ACTB-F: TAGCACAGCCTGGATAGCAA, and ACTB-R: TGACCCAGATCATGTTTGAGA) were used for PCR on a LC480 Real Time PCR system (Roche Pharma, Basel, Switzerland).

## Results

### Subjects’ characteristics

We studied the gene expression array of a cohort of 6 healthy, full-term neonates and 6 healthy adults with an equal gender distribution (33% female and 67% male).

In order to ratify the microarray findings, 16 neonates and 16 adults were selected to serve as the validation cohort. Gender distribution in the validation groups was 43.8% females vs. 56.3% males in the neonate group and 50% females vs. 50% males in the adult group. To note, 4 of these 16 samples were also used for the array. In both groups, available blood count data are shown on **S1 Table**.

### Platelet purity and RNA quality

**Fig 1:**
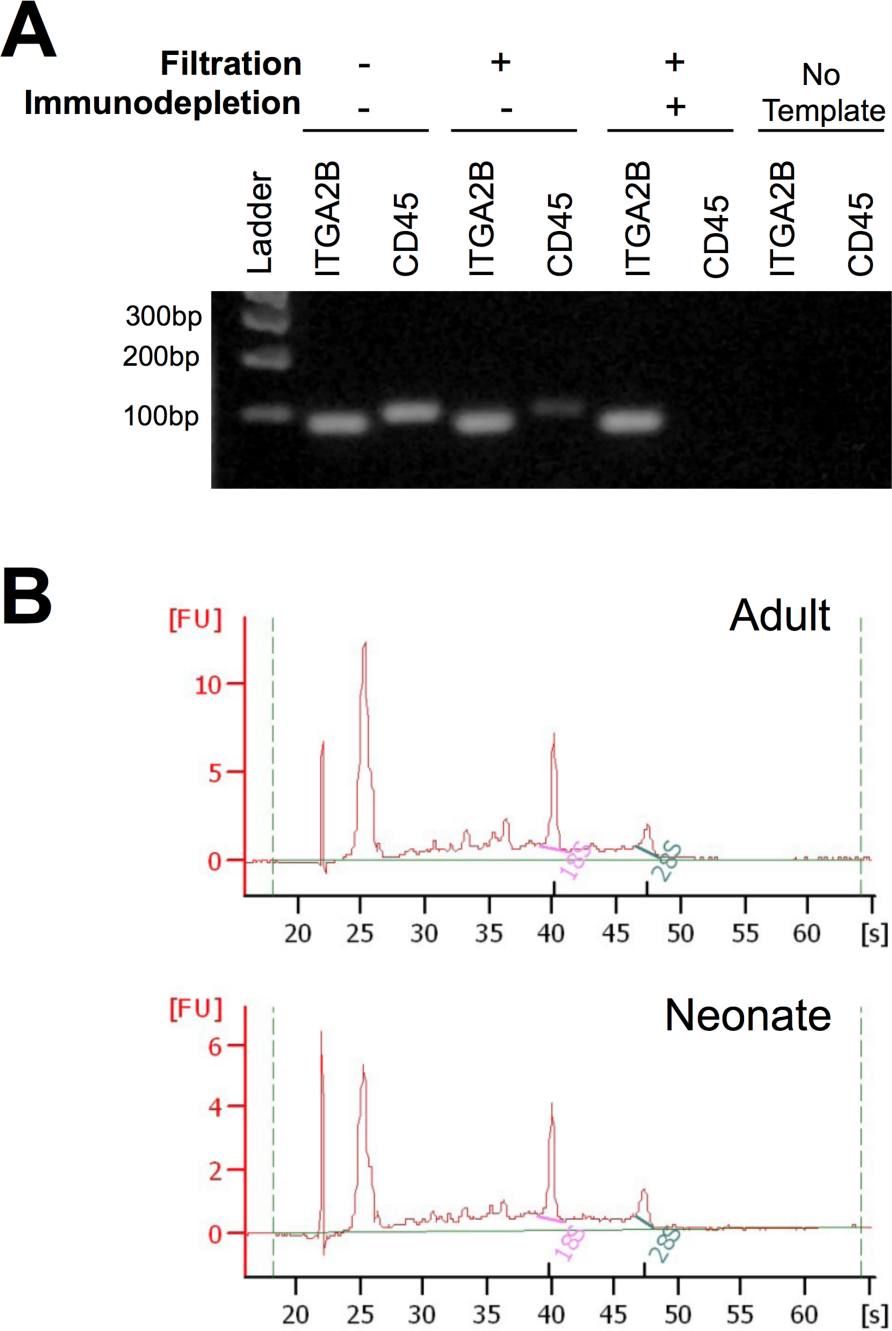
RNA integrity and purity in platelet samples. **(A)** Platelet content and leukocyte contamination in Platelet Rich Plasma (PRP) samples were evaluated by qRT-PCR amplification (40 cycles) of *ITGA2B and CD45*, respectively. PCRs were electrophoresed in an agarose gel. Both blood-derived PRP (lane 2) and filtered, but non-immunodepleted PRP (lane 4) exhibited *CD45* gene product amplification, indicating leukocyte contamination. In contrast, negligible *CD45* contamination was observed in filtered and immunodepleted PRP (ultrapure platelets) (lane 6)**. (B)** Bioanalyzer representative assessment of RNA in samples from ultrapure adult and neonate platelets demonstrating appropriate RNA quality and integrity.

Our highly efficient platelet purification protocol includes leukocyte reduction filtration and CD45 immunodepletion and is illustrated in **Fig 1A**. As shown, qRT-PCR assays using *ITGA2B* and *CD45* specific primers confirmed the presence of platelets and negligible leukocyte contamination, respectively, in the ultrapure platelet preparations. The quality of the RNA obtained was comparable in both neonatal and adult samples (**Fig 1B**).

#### Microarray platform comparative analysis

**Fig 2:**
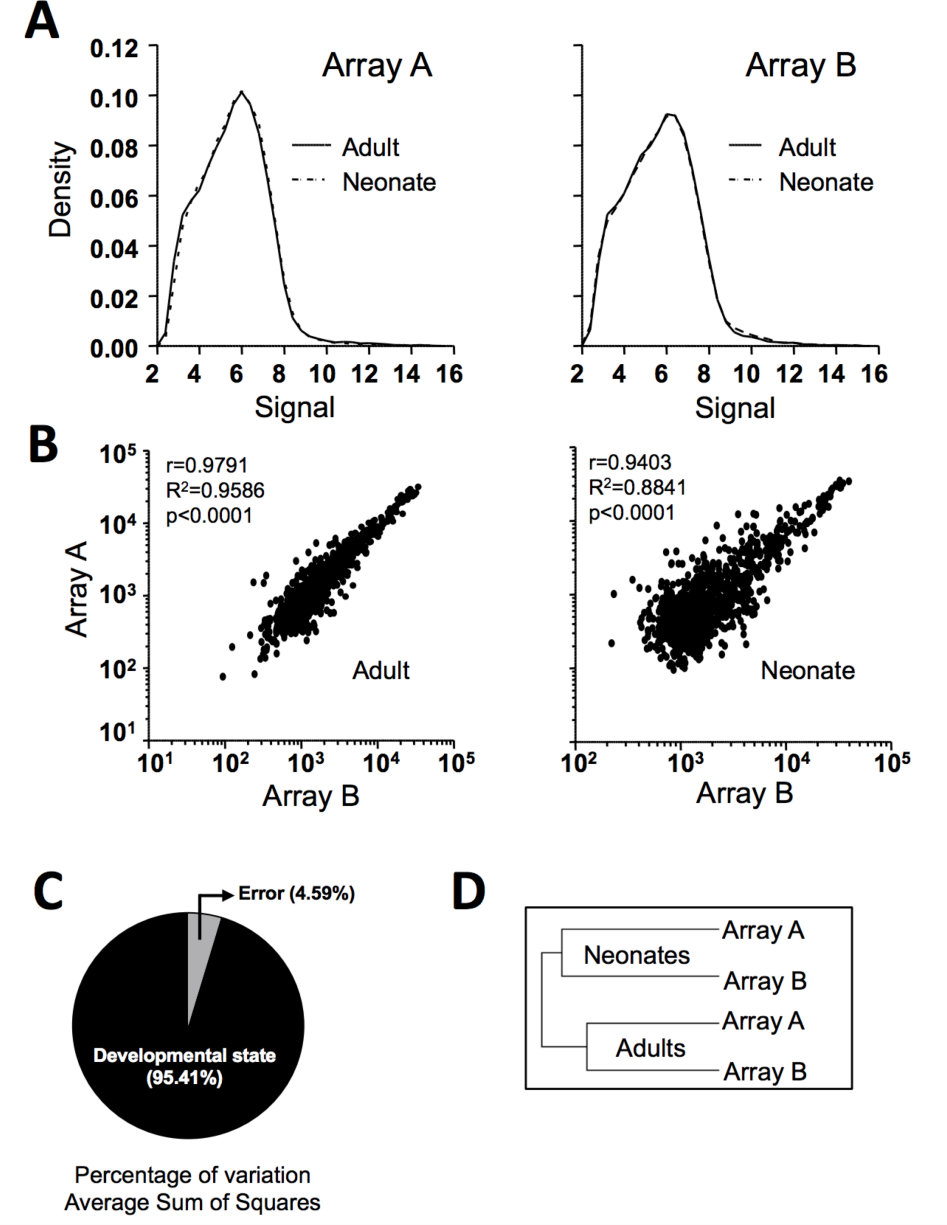
Microarray data comparison. **(A)** Density plot *vs*. Signal Intensity (processed data, log-2 scale) for two microarrays (A and B), each performed at an independent laboratory: LC Sciences laboratories (Houston) and the University of Murcia. **(B)** Scatter plots showing positive correlation between both arrays for each sample type (neonatal or adult platelets). **(C)** Source of variation expressed by signal to noise ratio of genes expressed at different developmental stages. **(D)** Hierarchical clustering dendrogram grouping samples by developmental stage.

Given the reduced number of neonate and adult samples included in the study and the statistical limitation, we aimed to increase the reliability of our findings by performing a genomic analysis in two independent laboratories (arrays A and B). As shown in **Fig 2A**, density plot analysis exhibited coincidence between adult and neonate samples for both arrays A and B, meaning that in both cases all the samples showed a similar gene signal distribution. Soundness of microarray data was also reflected in the dispersion graphs that indicate high correlation between arrays A and B for both adult and neonate samples (**Fig 2B**). Accordingly, Venn diagrams (**S1 Fig**) revealed that 857 out of the 1000 most highly expressed probe sets in adults and 683 out of the 1000 most highly expressed in neonates coincided on both platforms. We also performed a source variation test confirming developmental stage as the main variation factor of our results (**Fig 2C**). An unsupervised, hierarchical clustering analysis was applied to all probe sets, which yielded a dendrogram where the samples cluster by developmental stage (**Fig 2D**), suggesting that the data were perfectly comparable, thereby attesting to the solidity of our statistical testing results.

### Comparative analysis of adult platelet transcriptome and other microarray studies

**Fig 3:**
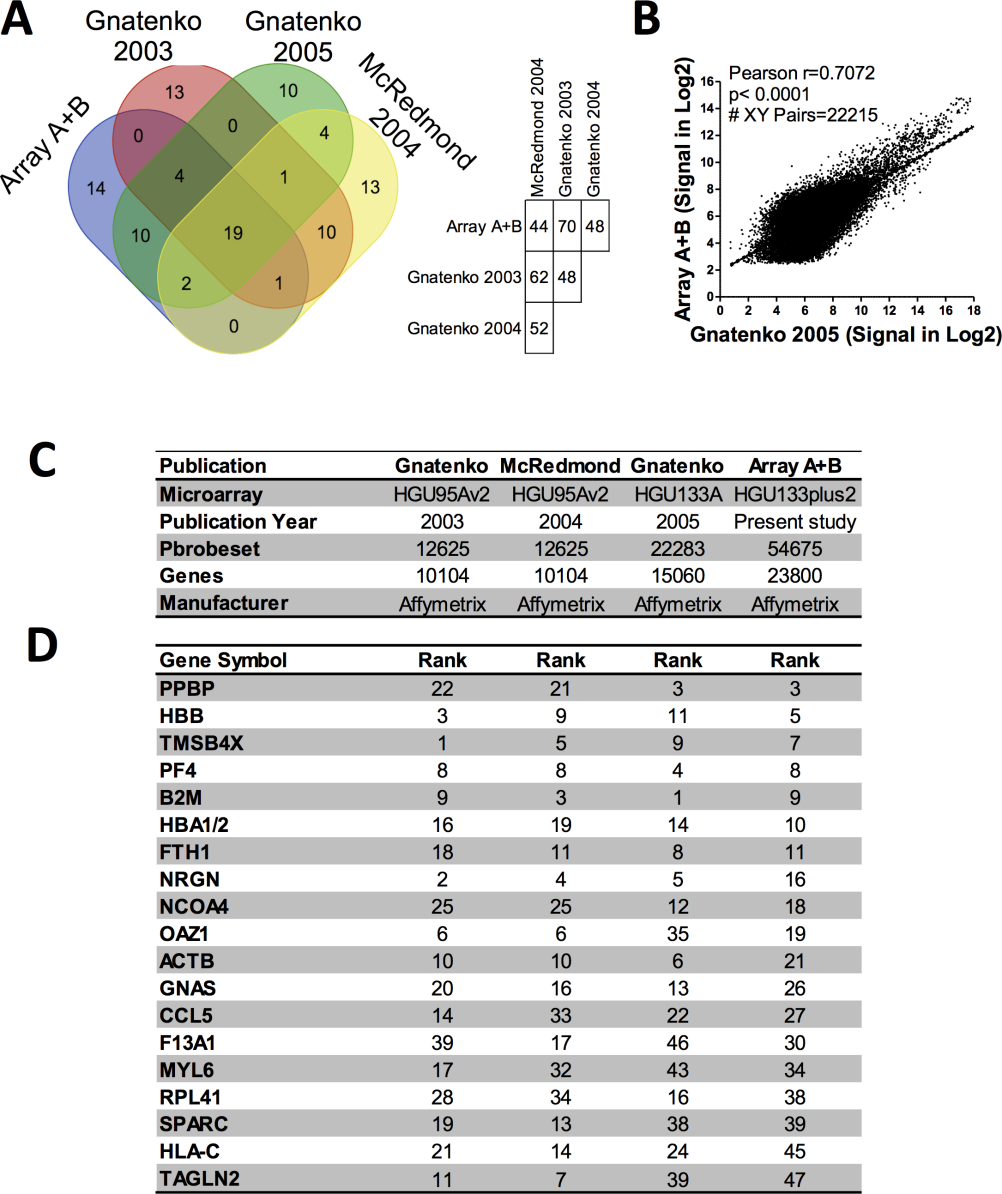
Adult platelet transcriptome comparison. **(A)** On the left, Venn diagram showing number of coincident genes among the 50 genes most expressed in the studies by Gnatenko 2003, McRedmond 2004, Gnatenko 2005 [23–25], and our array. On the right, a table showing the percentage of coincidence among the 50 most highly expressed genes in Gnatenko 2003, McRedmond 2004, Gnatenko 2005, and our array. **(B)** Scatter plots showing positive correlation between the Gnatenko 2005 study [23] and our array. Data are represented as processed intensity data (log-2 scale)**. (C)** Brief description of the microarray used for the comparison. **(D)** List of 19 genes present on all these different platforms, ranked by our array expression data.

To test the consistency and robustness of our transcriptome findings in adult platelets, we compared our results with those previously reported that used the same platform, albeit a different microarray version. Gnatenko’s study in 2005 [23] was selected for this comparison because they used a microarray, HU133A GeneChip (Affymetrix, Santa Clara, CA), pertaining to the same array series as the one run in our study (GeneChip® HU133 Plus 2.0 Array), as well as, because all probe sets present in that study (22,283) are also represented in the present study that contains 54,675 probe sets. Strikingly, on comparison of our adult results with the Gnatenko *et*. *al*. 2005 study in healthy controls, we find a 70% coincidence in the 50 most highly expressed genes (**Fig 3A**). This level of agreement corroborates the validity of our data and verifies the reproducibility of this technique. For an unbiased comparative analysis, we also carried out a free cut-off approach, comparing all the probes in common between Gnatenko’s study [23] and our array; specifically, 22,215 probes (removing internal Affymetrix probe controls). We obtained similar results (r = 0.7072; *p*-value<0.0001) after performing a Pearson’s correlation analysis (**Fig 3B**).

We then compared our adult data with two other arrays, i.e. the studies by Gnatenko in 2003 and by McRedmond in 2004 [24, 25]; both using the HG-U95v2 microarray version (with 10,000 genes analyzed) (**Fig 3C**). The top 50 expressed genes in the four arrays are presented in **S2 Table.** Overall, in comparison with previous platelet studies, our microarray data for adult platelets exhibited 44–70% coincidence with the 50 most highly expressed genes (**Fig 3A, right panel**). Indeed, of the 50 most highly expressed platelet genes in adults, 19 coincided in all microarrays (**Fig 3A and 3D**). Not surprisingly, some of these common genes encode for erythroid-derived proteins, as previously reported by those authors [24, 25].

### Gene expression profiling of neonatal and adult platelets reveals common highly expressed genes

**Fig 4:**
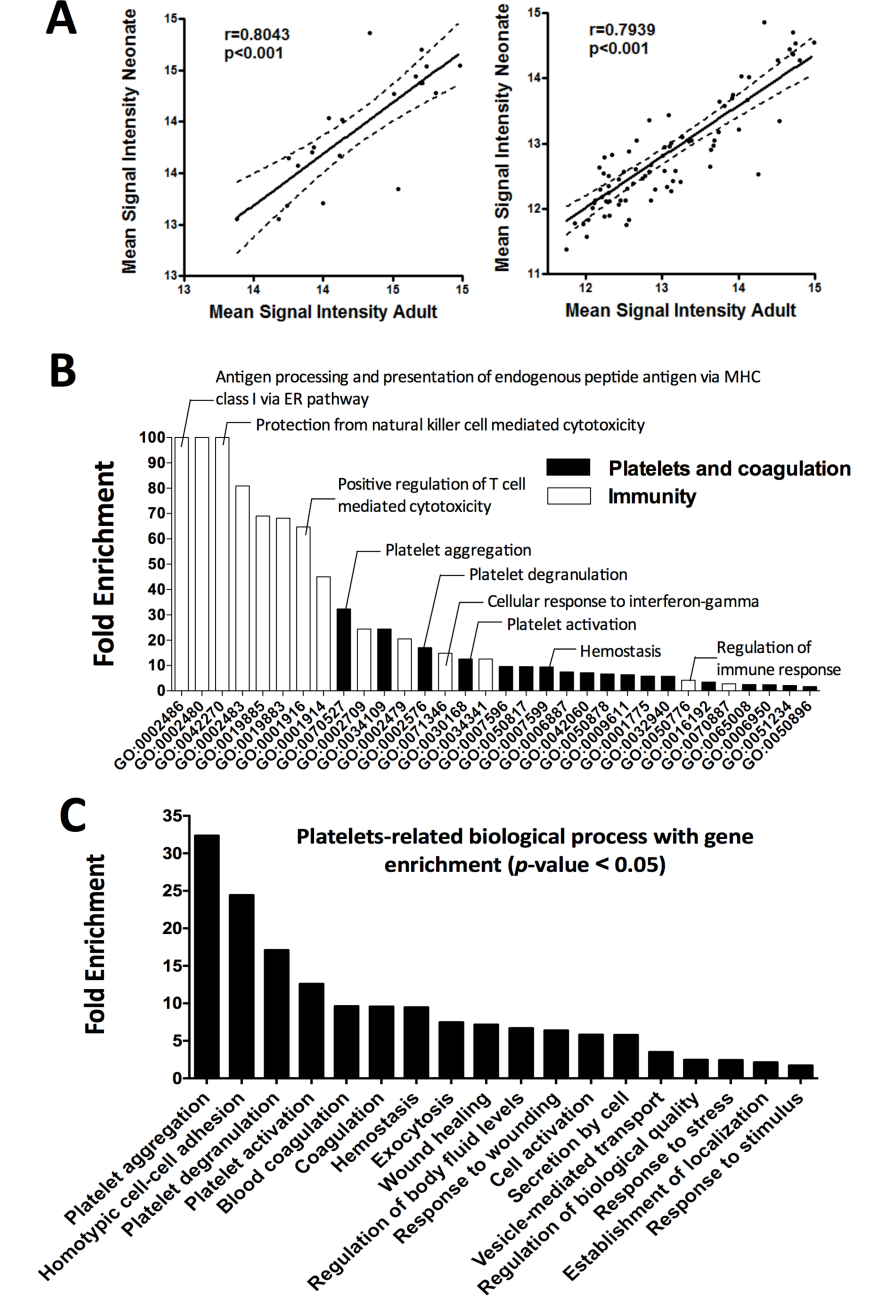
Correlation and gene enrichment analysis of genes highly expressed in both neonatal and adult platelets. **(A)** Dispersion graph illustrating the Pearson positive, significant (p<0.001) correlation between neonates (Y-axis) and adults (X-axis) using the top 25 **(left panel)** or the top 100 coincident most highly expressed platelet genes **(right panel). (B)** Gene enrichment analysis ranked by fold enrichment. Biological processes related with platelet function or immunity is displayed in black and white bars, respectively. **(C)** Platelet-related processes with gene enrichment, ranked by fold enrichment.

First, ranking the most highly expressed transcripts in neonatal and adult platelets revealed 21 coincidences (84%) among the 25 most highly expressed genes at both developmental stages, with excellent correlation in mRNA expression levels of these genes between adults and neonates (r = 0.8043, p<0.001) (**Fig 4A, left panel**). When extending the neonate *vs*. adult comparison to the 100 most highly expressed genes (**S3 Table**), we continued to see a very high percentage of gene coincidence (79.4%, p<0.001) and strong correlation in gene expression (**Fig 4A, right panel**).

Second, the biological relevance of the genes found to be highly expressed in both neonatal and adult platelets was explored by gene enrichment analysis. As summarized in **Fig 4B**, many genes involved in platelet function and immunity, or both, were present. Thus, highly expressed genes in platelets included *GNAS*, *ACTG1*, *ACTB*, *VCL*, *SPARC*, *SRGN*, *PPBP*, *F13A1*, *TMSB4X*, *and PF4* (**S4 Table**), which are enriched in key processes of platelet biology such as aggregation, degranulation, activation, and exocytosis (**Fig 4C**). Furthermore, we identified a few genes that play a general role in cell activation, but that are not restricted to platelets, such *CCL5 or B2M* (**S4 Table**). Not surprisingly, and taking into account the growing evidence of platelet participation in host defense, we observed that genes that take part in immune response processes, such as *HLA-A*, *HLA-B*, *HLA-C*, *HLA-E*, and *B2M*, are highly expressed in platelets. These genes are known to play a part in antigen processing and the presentation of exogenous and endogenous peptide antigens via MHC class I or T cell mediated cytotoxicity regulation, among others (**S4 Table**).

### Genes differentially expressed in neonatal *vs*. adult platelets

**Fig 5:**
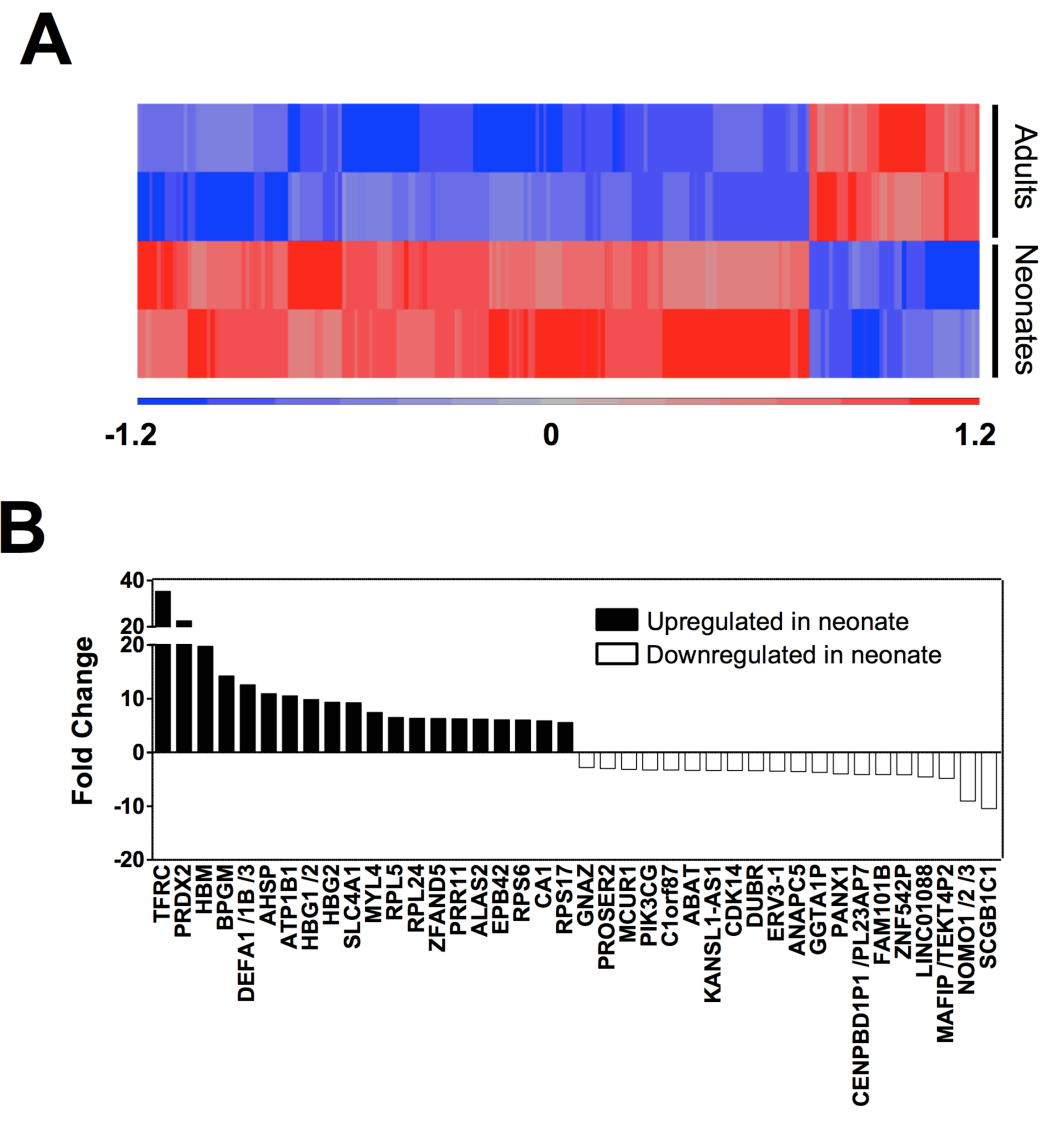
Genome profiling characterization of platelet during development. **(A)** Hierarchical clustering of differentially expressed genes between newborn and adult human platelets (FCh2, p<0.05). Samples grouped according to developmental stage are in rows and individual genes are shown in columns. Up-regulated genes having positive values are displayed in red and down-regulated genes with negative values in blue. **(B)** Top 40 differentially expressed genes (p<0.05) ranked by fold change. Neonate up-regulated genes have positive values and are displayed as black bars, while down-regulated genes, having negative values, are shown as white bars.

To identify platelet genes that are differentially expressed during ontogeny, we filtered gene expression by (i) p-value <0.05 and (ii) Fold-change >2. Thus, we identified 201 genes that are differentially expressed in newborn and adult platelets, 162 of which were up-regulated and 39 were down-regulated (**S5 Table**). A heat map of these differentially expressed genes is presented in **Fig 5A.** Focusing attention on the top 20 up-regulated genes in neonatal platelets (**Fig 5B**), we noticed that they can be divided into two main groups: one group coding for erythroid-derived proteins (*TFRC*, *BPGM*, *AHSP*, several *HBs* genes, *SLC4A1*, *ALAS2*, *EPB42*, *CA1*) and the one comprised of genes involved in protein synthesis and degradation, such as ribonucleoproteins (*RPL5*, *RPL24*, *RPS6*, *RPS17*) or the ubiquitin-proteasome system (*ZFAND5*) (**Fig 5B**). Of note, the second most significantly over-expressed gene in neonatal platelets (fold change of 22 *vs*. adult platelets) is *PRDX2*, an antioxidant enzyme linked to platelet reactivity [26]. Moreover, some of the most differentially overexpressed genes during ontogeny (fold change >7) are those involved in cytoskeletal reorganization such as *MYL4* [27].

As far as the most under-expressed genes in neonatal platelets are concerned, they include genes involved in calcium transport or metabolism (*MCUR1*) [28], actin cytoskeleton reorganization *(FAM101B)* [29], and cell signaling (*GNAZ*, *PANX1*) [30, 31] **(Fig 5B).**

It is well known that neonatal platelets are hyporeactive to epinephrine, as they express α-adrenergic receptors less [11]. Accordingly, *ADRA2A* expression levels in our array were 1.9-fold lower in neonatal compared to adult platelets. However, probably due to the reduced number of samples, the *p*-value (p = 0.07) was above the threshold for 'significance' (p<0.05). We therefore extended the list of genes differentially expressed even further to include those transcripts with a highest difference in fold change but *p-*values ranked 0.05–0.15 **(Table 1).** Applying this *p*-value criterion, neonatal platelets again revealed enrichment of genes involved in DNA/mRNA processing (*HMGB2*) or cytoskeletal reorganization (*TUBB2A)* and under-expression of genes encoding calcium-binding proteins (*C17orf57*, *CABP5)* and granule exocytosis (*RTN1)*.

**Table 1:** 20 most under- and over-expressed genes in neonatal platelets.

Gene Symbol	Fold Change	*p*-value
GYPA	23.3	0.07
S100A8	12.1	0.06
YOD1	11.6	0.14
B3GNT2	11.4	0.12
DLK1	10.9	0.05
TUBB2A	10.5	0.05
HMGB2	9.8	0.06
RHAG	9.4	0.12
CAT	7.7	0.13
HSPD1	7.3	0.11
NPM1	7.1	0.09
LXN	6.9	0.10
RPL35	6.2	0.05
FECH	6.0	0.13
TMEM110-MUSTN1	5.7	0.06
HSPA8 /// SNORD14C-D	5.5	0.08
CCNB1	5.4	0.12
GSPT1	5.3	0.06
RPS21	5.2	0.12
GCH1	5.2	0.08
MOB1A	-3.2	0.08
RAB30	-3.2	0.13
PLA2G12A	-3.4	0.13
RTN1	-3.5	0.06
NREP	-3.7	0.12
CABP5	-3.8	0.14
C17orf57	-3.8	0.07
ITGB3BP	-4.1	0.12
OVOS2	-4.2	0.06
HLA-DPB1	-4.3	0.08
ENDOD1	-4.5	0.08
MSRB3	-5.0	0.06
ZBTB38	-5.0	0.13
TMEM70	-5.5	0.13
BEND2	-5.7	0.09
KIAA0564	-6.3	0.13
LOC100129973	-6.6	0.06
SLC25A43	-7.0	0.12
ZNF385D	-7.8	0.08
GRHL1	-8.1	0.08

*p*-value range of 0.05–0.15. Ranked by fold change.

### Validation of gene expression array data

To validate the findings of the microarray analysis of neonatal and adult platelet pool samples, we performed a series of qRT-PCR experiments in a new set of samples (n = 16 samples/group) to quantify the mRNA transcripts that appeared differentially expressed during development in our microarray study. In particular, we analyzed *ADRA2A*, encoding for the epinephrine receptor, *GNAZ*, *GNB5*, *PI3KCG*, *RPL32*, *and PRDX2* expression.

**Fig 6:**
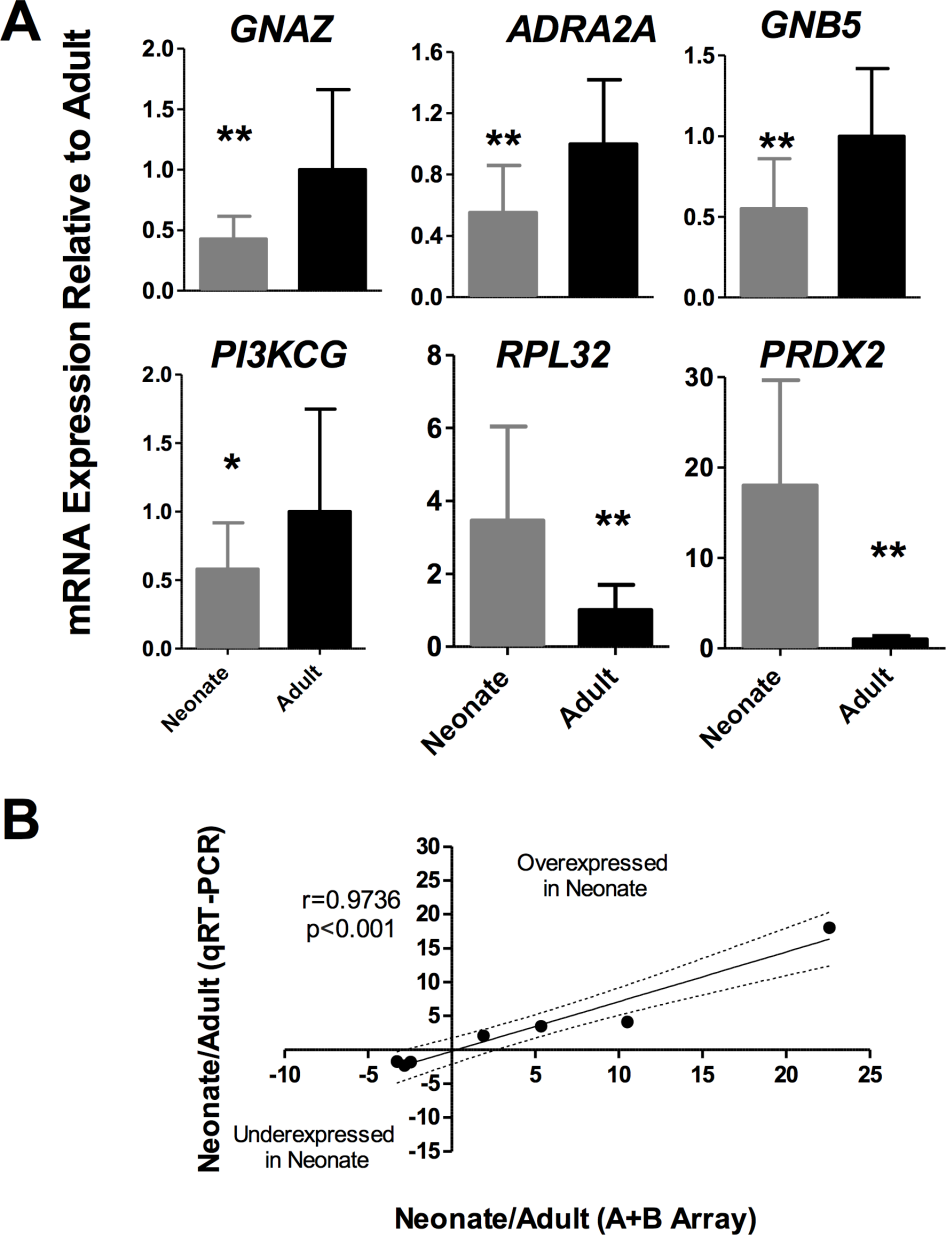
Real-time quantitative PCR validation of mRNA levels. (A) N = 16 samples per group. Values represent Relative Mean Expression ± SD (as a percentage of adult control platelets) of each mRNA normalized to *ACTB* (β-Actin) (*p<0.05; **p<0.01). (B) Spearman correlation between fold change obtained in A+B array and fold change obtained by qRT-PCR in the validation cohort.

The qRT-PCR assays were consistent with our microarray data and demonstrated a two-fold reduction in *GNAZ*, *ADRA2A*, *GNB5*, and *PI3KCG* mRNA in neonatal *vs*. adult platelets (p<0.01, **Fig 6A**). Immunoblotting further validated the underexpression of *GNAZ* at the protein level (p<0.05, **S2 Fig**). On the other hand, we confirmed that *RPL32* and *PRDX2* were up regulated in neonates compared to adult (p<0.01, **Fig 6A**). Interestedly, all mRNA expression levels assessed by qRT-PCR were highly correlated with data obtained in array (r = 0.9736; p<0.001 **Fig 6B**).

### Biological pathway analysis

We applied the Partek Genomics Suite and Partek Pathway analysis linked to KEGG database to the list of differentially expressed genes (p<0.05 and Fold Change>2) in an attempt to identify biological pathways that may exhibit major functional differences during development. **Table 2**lists the biological pathways obtained from this analysis sorted by enrichment score, p-value, and % of genes of the pathway that are present. As shown, most of these pathways having potentially different functional status in neonates *vs*. adults, are related to mRNA processing, as well as protein synthesis and degradation.

**Table 2:** Signaling Pathways most differentially represented between newborn and adult platelets.

Pathway Name	Enrichment Score	Enrichment p-value	% genes present	Genes
Ribosome	13.3	1.67E-06	21.97	MRPL14, MRPS15, MRPS16, RPL5, RPL10A, RPL13, RPL14, RPL18A, RPL21, RPL22, RPL24, RPL26L1, RPL27A, RPL32, RPL34, RPL35A, RPL36, RPL36AL, RPL37, RPL37A, RPL38, RPS4X, RPS6, RPS10, RPS15A, RPS17, RPS17L, RPS28, RPSA
Proteasome	8.3	2.38E-04	27.27	PSMA4, PSMB1, PSMB2, PSMB4, PSMC1, PSMC5, PSMD1, PSMD4, PSMD8, PSMD12, PSME4, SHFM1
p53 signaling pathway	5.4	4.69E-03	19.12	BID, CCNE1, CCNG2, CHEK2, GADD45B, MDM4, PTEN, RRM2, SERPINE1, SESN3, SFN, THBS1, TNFRSF10B
Spliceosome	4.9	7.36E-03	15.38	ALYREF, DDX46, DHX16, HNRNPA1, HNRNPM, ISY1, LSM5, NCBP1, PRPF18, PRPF38A, RBMXL1, SF3B1, SLU7, SNRPA1, SNRPE, SNRPG, SRSF5, SRSF10, THOC2, U2SURP
Cell cycle	4.8	8.50E-03	15.45	ABL1, ANAPC1, ANAPC5, ANAPC10, ANAPC11, BUB1B, CCNE1, CDC14A, CDC14B, CDC27, CDKN1C, CHEK2, E2F2, FZR1, GADD45B, MCM6, SFN, SMC3, TGFB2
mRNA surveillance pathway	3.9	2.09E-02	15.56	ALYREF, CPSF2, CPSF6, CSTF3, GSPT2, MSI2, NCBP1, NXT1, PABPC1, PABPC3, PNN, PPP2R1B, PPP2R5E, RNGTT
RNA transport	3.2	3.93E-02	12.88	ALYREF, CLNS1A, EEF1A1, EIF1, EIF2S3, EIF3B, EIF4G3, FXR1, GEMIN8, KPNB1, NCBP1, NDC1, NUP43, NUP50, NUP153, NUPL1, NXT1, PABPC1, PABPC3, PNN, THOC2
Ubiquitin mediated proteolysis	3.2	4.03E-02	13.33	ANAPC1, ANAPC5, ANAPC10, ANAPC11, CDC27, CDC34, CUL2, CUL5, DET1, FZR1, HERC3, HERC4, SOCS3, TRIM32, UBA6, UBE2NL, UBE2O, XIAP

Similar outcomes were reached with additional enrichment analyses using the PANTHER Overrepresentation Test that examined samples by biological process, removing the most highly expressed genes in cord blood reticulocytes [32] and filtering results by p<0.001 (**S6 Table)**.

## Discussion

This study provided the first comprehensive comparison of transcriptomes of human neonatal and adult platelets. We used microarray expression tools in a few pooled samples of ultra-pure, leukocyte- free neonatal and adult platelets. Despite the limitation of the small sample for statistical purposes, the use of pools aids in homogenizing results within each sample group. In fact, it has been reported that inference for most genes is not adversely affected by pooling samples prior to hybridization [33]. In line with these observations, our original array data were highly consistent with those obtained using a different set of pooled samples. There was also a high agreement among the expression profiles attained by two independent laboratories. The robustness of the procedure was further endorsed by our finding of approximately 70% coincidence among the top 50 most highly expressed genes in adult platelets in our microarray study and those recently reported by another research group [23] using a similar chip (HGU133Av2 and HGU133A [23], respectively). The degree of coincidence is lower when we compared our data with older studies [24, 25] that applied a different array version (HGU95Av2).

Probably as a reflection of its precursor, the MK, we found a high correlation between neonate and adult platelet transcriptomes. Thus, the gene expression profiling of MKs throughout ontogeny has revealed a close relationship between two contiguous stages: human embryonic-derived stem cells and fetal liver-derived MKs, and between neonate-derived and adult-derived MKs. Thus, the changes in the transcriptome analysis between neonate-derived and adult-derived MKs tended to be modest [9]. Accordingly, our study found that many of the mRNAs are highly expressed and conserved during ontogeny. Therefore, both adult and neonatal platelet transcriptomes exhibited an abundance of mRNAs involved in platelet function, such as structural proteins (*TUBB1*, *ACTB*, *VCL*), cytoskeleton regulators (*SH3KBP1*, *TMSB4X*), platelets chemoattractant proteins (*PF4*, *PPBP*), and signaling proteins (*NRGN*, *RGS18*, *RGS10*, *GNAS*) **(S3 Table)**. Among those genes, previous reports found *SRGN*, *PPBP*, *F13A1*, and *PF4* to be highly expressed in mammals [34]. Worthy of special note is *PPBP* (Pro-Platelet Basic Protein), which encodes a platelet-derived growth factor of the CXC chemokine family. This protein is highly expressed during megakaryopoiesis; it is stored within α-granules, and it activates neutrophils prior to secretion through CXCR2 [35]. PF4 is another protein that is conserved throughout development and is abundant in platelet α-granules. In addition to its well-known function in hemostasis, PF4 may also exert a pro-inflammatory effect in the presence of other stimuli, such as TNF-α, leading to neutrophil activation [36].

Although the role of platelets in hemostasis is now well understood, their role in inflammation and immune response has yet to be fully elucidated [37, 38]. Remarkably, in both adult and neonatal platelets, our gene enrichment study demonstrated the abundance of mRNAs translating proteins involved in immune response. Thus, the top 100 most highly expressed genes in adult and neonatal platelets included genes having to do with processing and peptide antigen presentation or with T-cell regulation or natural killer mediated cytotoxicity, such as beta 2 (*B2M*) and Human Leukocyte Antigens (*HLA-A*, *-B*, *-C*, and–*E)*. These findings further support this increasingly recognized role of platelets in host immune defense [39, 40].

In addition to demonstrating that the expression of many genes in platelets is preserved throughout development, our study has also identified 200 genes that are differentially expressed in neonatal and adult platelets. Interestingly, among the significantly up-regulated RNA transcripts in infants, the most prevalent are those which are present in biological pathways related to protein synthesis, trafficking, and degradation, such as ribosome, proteasome, and spliceosome, among others (**Tables 2 and S6**). This huge divergence in protein synthesis machinery between neonatal and adult platelets is most certainly puzzling. Platelets are now recognized as being capable of “*de novo”* protein synthesis, particularly when activated [41, 42]. Interestingly, a recent study has shown that murine neonatal platelets have a longer lifespan than adult platelets [16]. While this finding cannot be directly extrapolated to humans, we can speculate that changes in platelet lifespan during development may be driven by the differential expression of genes involved in the protein metabolism pathways that we have observed in this study. Thus, our analysis showed that *JunD*, which has been proposed to protect cells from p53-dependent senescence and apoptosis [43], is overexpressed in neonatal platelet (4.76-fold, p-value<0.01). Moreover, proteasome inhibition, one of the most different pathways found in our study, has recently been shown to shorten platelet lifespans [44].

Finally, our results provide new insights to better understand the well-established hyporeactivity of neonatal platelets. We have identified genes that are up or down-regulated in neonatal platelets and that relate directly or indirectly relationship with platelet reactivity. As for up-regulated transcripts, *PRDX2* (peroxiredoxin 2) was found in the array study to be substantially up-regulated in neonatal platelets (22.59-fold, p-value<0.01) and was demonstrated further in our validation cohort **(Fig 6)**. *PRDX2*-deficient murine platelets displayed increased adhesion and aggregation on collagen stimulation [45]. Consequently, overexpression of peroxiredoxin 2 may play a role in impaired collagen-mediated platelet activation, which is known to be defective in neonatal platelets [13]. Concerning underexpressed genes in newborn platelets, they mainly participate in endo- and exocytosis, cell signaling, protein transport, and calcium metabolism/transport, among others. First at all, granule secretion is one process seen to be flawed in neonatal platelets [14, 15]. Furthermore, we have found a tendency toward lower expression of the SNARE protein *VAMP7* (-4.33-fold), the depletion of which leads to a partial defect in granule exocytosis [46] and *RTN1* (reticulon 1, -3.48-fold), which is able to interact with SNAREs proteins and thought to be involved in exocytosis in neuroendocrine cells [47]. Secondly, our findings show genes involved in cell signaling (i.e. *GNAZ*, *GNB5*) to be underexpressed in neonatal platelets. Most platelet agonists, except collagen, induce activation through G-protein coupled receptors (GPCR). Our array and the subsequent validation study show that both *ADRA2A* and *GNAZ* are underexpressed in neonatal platelets. This probably accounts for neonatal platelets’ exceedingly decreased response to epinephrine [11, 48]. Additionally, we found down regulation of several genes, such as *C1orf87 or MCUR1*, that are related to with calcium homeostasis, a well-known defective pathway in newborn platelets [49]. Finally, the underexpression of *ITGB3BP* and other *RUNX1*-regulated genes due to *RUNX1* mutations, can cause thrombocytopenia or impaired platelet aggregation and secretion [50]. Although *RUNX1* expression levels were not different throughout development, our results showed a tendency toward lower expression in some *RUNX1*-regulated genes, for instance, *CABP5* (-3.82-fold), *RABGAP1L* (-2.93), *CXCL5* (-2.85), *EPB41L3* (-2.65), and *SLC24A3* (-2.29).

In summary, this study has been the first to demonstrate variations in platelet transcriptome throughout ontogeny. Our results suggest that differential gene expression could contribute to changes in platelet reactivity and/or platelet lifespan during development. Moreover, this new survey of the mRNA profile in human platelets further corroborates the emerging role of platelets as immune cells. Future investigation is required to determine whether these platelet transcriptome differences are a reflection of differences at the megakaryocytic level and to further evaluate the potential hemostatic and non-hemostatic consequences of these developmental differences in platelet gene expression.

## Supporting information

S1 Fig Venn diagram of the coincident probe sets among the 1000 most highly expressed probe sets between both arrays and for each type of sample (neonate *vs* adult). (TIF)

S2 Fig Quantification of GNAZ protein levels (Gαz) measured by immunoblotting and analyzed by ImageJ. Western blot pictures depicted here are representative images of a minimum of 11 samples per group. Values represent Relative Mean Expression ± SD (as a percentage of adult control platelets) of each mRNA or protein normalized to β-Actin (*p<0.05).
(TIF)

S1 Table Blood counts in neonates and adult samples. (XLSX)

S2 Table The top 50p platelet genes most highly expressed in previous works by Gnatenko, McRedmond, and A+B microarrays and their rank in each array. Coincident genes in all microarrays are marked in bold.
(XLSX)

S3 Table Coincident genes among the 100 most highly expressed in adult and neonatal platelets. Columns represented the A+B combination microarray signal expressed in Log2 and their rank at each developmental stage (neonate and adult), sorted by adult rank.
(XLSX)

S4 Table Gene enrichment analysis using the coincident genes in the 100 most highly expressed during ontogeny (in both adult and neonatal platelets). Significance was taken as *p*<0.05.
(XLSX)

S5 Table List of differentially expressed platelet genes throughout development. ANOVA results were filtered by more than 2-fold change and a significance level of *p*<0.05 between adults and neonatal platelets.
(XLSX)

S6 Table Gene enrichment analysis using the platelet genes most Differentially expressed between adults and neonates, but removing highly expressed genes in reticulocytes. Significance was taken as p<0.001
(XLSX)
