# Supplementary figures and images for: Comprehensive comparison of neonate and adult human platelet transcriptomes
Source: PLoS One. 2017 Aug 16;12(8):e0183042. doi: 10.1371/journal.pone.0183042 (PMC5559076; doi:10.1371/journal.pone.0183042)

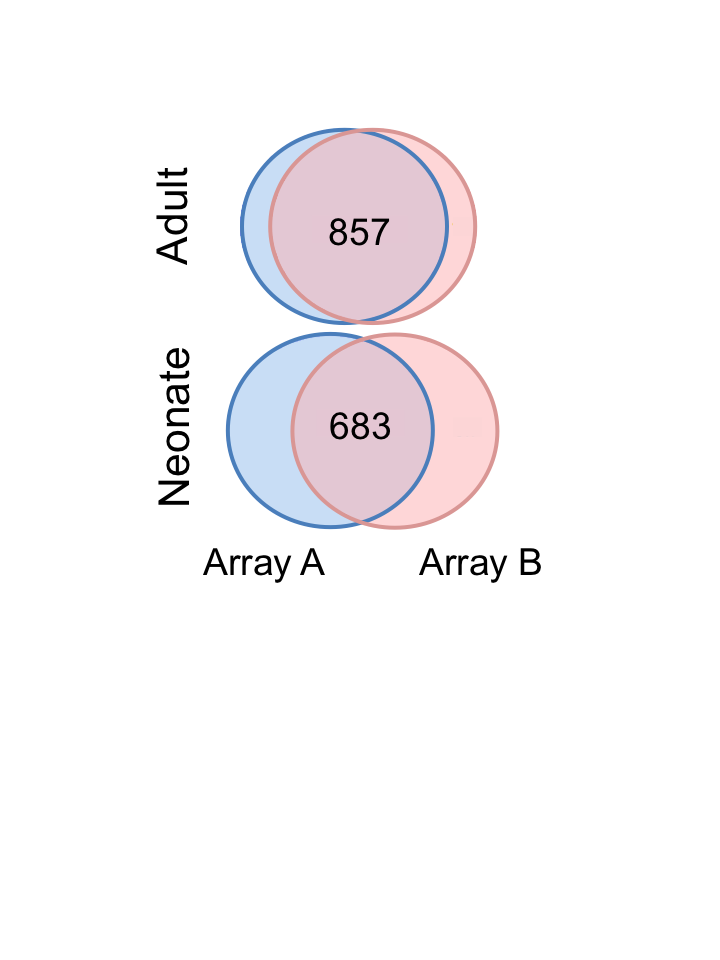

Supplement: S1 Fig — (TIF) [file pone.0183042.s001.tif]
